# Breakfast: a multidisciplinary approach

**DOI:** 10.1186/1824-7288-39-44

**Published:** 2013-07-10

**Authors:** Antonio Affinita, Loredana Catalani, Giovanna Cecchetto, Gianfranco De Lorenzo, Dario Dilillo, Giorgio Donegani, Lucia Fransos, Fabio Lucidi, Chiara Mameli, Elisa Manna, Paolo Marconi, Giuseppe Mele, Laura Minestroni, Massimo Montanari, Mario Morcellini, Giuseppe Rovera, Giuseppe Rotilio, Marco Sachet, Gian Vincenzo Zuccotti

**Affiliations:** 1 Italian Parents Movement (MOIGE), Milan, Italy; 2 National Association of Italian Pedagogists (ANPE), Milan, Italy; 3 Italian National Association of Dietitians (ANDID), Milan, Italy; 4 Department of Paediatrics, University of Milan, “Luigi Sacco” Hospital, Milan, Italy; 5 Food Education Italy, Milan, Italy; 6 Italian Association of Food Science Specialists (ANSISA), Milan, Italy; 7 Department of Psychology of Developmental and Socialisation Processes, University “La Sapienza”, Rome, Italy; 8 Censis Cultural Policies Sector, Milan, Italy; 9 Italian Federation of Paediatric Doctors, Milan, Italy; 10 Department of Communication and Social Research, University “La Sapienza”, Rome, Italy; 11 Director of the European Masters Course “History and Culture of Food”, University of Bologna, Bologna, Italy; 12 Rome University “Tor Vergata”, Rome, Italy; 13 Italian Institute of Packaging, Milan, Italy

**Keywords:** Breakfast, Dietary choices, Diet, Multidisciplinary approach, Nutrition

## Abstract

**Background:**

The role of breakfast as an essential part of an healthy diet has been only recently promoted even if breakfast practices were known since the Middle Age. The growing scientific evidences on this topic are extremely sector-based nevertheless breakfast could be regarded from different point of views and from different expertises. This approach, that take into account history, sociology, anthropology, medicine, psychology and pedagogy, is useful to better understand the value of this meal in our culture. The aim of this paper was to analyse breakfast-related issues based on a multidisciplinary approach with input by specialists from different fields of learning.

**Discussion:**

Breakfast is now recommended as part of a diet because it is associated with healthier macro- and micronutrient intakes, body mass index and lifestyle. Moreover recent studies showed that breakfast improves cognitive function, intuitive perception and academic performance. Research demonstrates the importance of providing breakfast not only to children but in adults and elderly too. Although the important role breakfast plays in maintaining the health, epidemiological data from industrialised countries reveal that many individuals either eat a nutritionally unhealthy breakfast or skip it completely.

**Summary:**

The historical, bio-psychological and educational value of breakfast in our culture is extremely important and should be recognized and stressed by the scientific community. Efforts should be done to promote this practice for the individual health and well-being.

## Background

The role of breakfast as an essential part of an healthy diet has been only recently promoted even if breakfast practices were known since the Middle Age. Breakfast has changed across time being largely dependent on culture and on the availability of traditional foods in different countries.

The recent growing scientific evidences about the value of breakfast are extremely sector-based looking at specific aspects or part of the overall of the topic, nevertheless breakfast could be regarded from different point of views and from different expertises. Beside the most and well studied nutritional aspects, socio-anthropological and bio-psycho-pedagogical attitudes should not be neglected and warrant consideration too.

This multidisciplinary approach, that take into account history, sociology, anthropology, medicine, psychology and pedagogy, is useful to better understand the value of this meal and highlights its importance in our culture.

The aim of this paper was to analyse breakfast-related issues based on a multidisciplinary approach with input by specialists from different fields of learning.

## Discussion

### Historical point of view

The Italian word “*colazione*” comes from the Latin *Collationes* (compilation, collection), a fifth century set of moral precepts and instructions on monastic life, an edifying work by Giovanni Cassiano, one of the fathers of medieval monasticism. Monks were obliged to remain silent during meals while one of them read aloud from religious texts: the most popular was the one by Cassiano; as a result, over the years the *Collationes* became associated with meals. The meal became the ultimate *collatio* which in turn led to the modern meaning of *colazione*.

The *Regula monachorum* written by St. Benedict, which provides detailed information about the daily organisation of monastic life, prescribes only two meals: lunch and supper. Since it makes no mention of a “first meal”, and considering that the *Regula* describes in detail the daily obligations and activities of the monks, no particular attention was presumably paid to this meal; this is true for medieval society at large [1]. We know very little about mealtimes except that generally speaking meals were eaten earlier than they are today, and that the working classes ate before the upper classes - an important sign of social distinction [2].

Even when the first meal of the day – lunch or “meal”– was preceded by a “break-fast”, now commonly written *breakfast* (the English word refers to a meal that broke the *fast*, in other words, the night-time fast), it was very similar to lunch, although this version was simpler and smaller in quantity and quality. In particular, it did not involve hot food; in Mediterranean Europe breakfast normally included salted meats and cheese accompanied by bread and wine.

In the eighteenth century, Giovanni Baretti, who used to write for the English, tried to explain certain characteristics of the Italian’s eating habits. Amongst other things he noted that compared to the British, in Italy nothing hot is given to children in the morning, because it is generally thought that hot food in the morning can ruin the teeth of young people and weaken their temperament [3]. Baretti wrote this in 1768 at a time when scientific ideas and research had revised medieval codes. Although the language is the same; the concept of “temperament” is old, and so is the separation of food into “hot” and “cold”, and “wet” and “dry”, which dates to the Hippocratic-Galenic tradition. As a result, the general “opinion” to which the eighteenth-century intellectual presumably refers was a leftover from the considerations elaborated centuries earlier in the scientific world and which later became common knowledge; this was a common occurrence in the history of ideas.

The novelties which began to emerge in food habits after the Enlightenment in the early eighteenth century were influenced by the introduction of colonial beverages in Europe: coffee, tea and chocolate, products from Asia (tea) or America (although the Europeans had been the first to bring coffee and cocoa from the Middle East and replant them in America) which maintained their exotic and elitist allure for many years. As a result, this new elitist breakfast model was in great demand by the upper classes, because it was hot and sweet, rather than cold and salty.

The dilemma of the Young Gentleman in the poem *Mattino* by Giuseppe Parini (1763) was whether to choose coffee or chocolate as soon as he woke, a decision he made each morning depending on how the fancy took him. In any case, the poet writes, it was indeed worthwhile – and here his satire is withering – “that seas be sailed, wars fought, men massacred, to bring said goods to his mouth” [4]. These were the new preferences and flavours of an aristocratic breakfast in eighteenth-century Italy [5]. In Spain, chocolate won the day, in France coffee. Instead in England and the Netherlands, tea triumphed thanks to unscrupulous marketing by trading companies, backed by the anything but disinterested support of some members of the medical world who were ready to assert the extraordinary effects of those beverages on people’s health (in much the same way French physicians supported the virtues of coffee) [6].

In his *Relazione degli usi e costume*, Giovanni Baretti is one of the few who also included breakfast in his observations about food habits (perhaps because he himself had experienced these important changes) [3]. In the first place he noted that the meal differs according to people’s age and health. As mentioned above, children were not given hot food or beverages, but simply bread, or bread and cheese, or bread and a seasonal fruit. Coffee and chocolate were eaten during the meal (breakfast) by civilised adults – a distinction which involved not only the person’s age but also his social class, since the word “civilised” corresponded to “the upper classes”. These customs were still not granted to peasants and ordinary people. In fact, Baretti goes on to say that most peasants and lower classes had porridge for this meal (breakfast) and when it was piping hot covered it in fresh butter and a few slices of cheese. Tea was not drunk by ordinary people, but neither was it drunk by the upper classes: only ladies sipped it, occasionally, to ward off colds. However, Baretti added that during his last trip to Italy he noted it had become very popular, especially in Italian maritime cities, and that many of his compatriots told him - with tactful displeasure - that the vanity of wanting to imitate the *milady* of England had begun to corrupt Italian ladies, as well as greatly increasing the introduction of this useless and expensive drug. Regarding the habits of a rich minority, Baretti refers that in the summer Italians get up early and in the morning love to sit in the cool of the countryside. Some have a country house where they spend the summer months. Other people often go to the fields at sunrise to have breakfast with the whole family; they take dry food, salted meats, cheese, fruit and wine, throw a tablecloth on the grass and have a hearty meal near a stream or a fountain, breathing in the flower-scented air. They return to the city before the sun becomes too fierce, and believe that this morning exercise is very healthy and extremely necessary for the children. He adds that on the whole city dwellers have not adopted this habit.

Together with colonial beverages, the invention of artificial cold during the industrial revolution made milk very popular because it could now be preserved (and not necessarily turned into cheese). Like coffee and chocolate, milk became a possible new protagonist of the European diet; however, like tea, chocolate remained a social watershed. On the contrary, over the years coffee became increasingly popular. A new “break-fast” model became feasible and gradually trendy – not only and not just because it was drunk at all hours – but because of the kind of food and beverages which were eaten. Purists in Italy continued to call it “*colazione*”, reiterating the traditional similarities between the two meals, differentiated only by the adjective “first” and “second”. In certain social milieu (especially the lower classes) this was true, and sometimes still is, especially because it involves more salty than sweet foods: in fact, this is a characteristic of breakfasts in many countries all over the world. Elsewhere the two models are different and justify the name change: “*colazione*” [breakfast] is only the morning meal, while the other “*colazione*” has been renamed lunch. All these changes are recent, and it would be well worth studying these food habits and linguistic traditions more in-depth.

### Socio-Anthropological point of view

The cultural importance of food has been neglected for years; only in the early seventies food began to spark the interest of social scholars. In fact, the consolidated *snobbism* towards anything which is part of our daily lives continued to weave its web of resistance, perhaps dictated by the major inferiority complex of social sciences which were trying to move up the ladder vis-à-vis more established scientific disciplines such as biology or medicine. For many years this complex made them value and concentrate on topics, such as economics or politics, which they considered worthy of attention *per se*.

The 1964 study by Lévi-Strauss “Le cru et le cuit” can be considered a watershed document. In his paper the famous anthropologist identified fire as a mediating element between man and nature; he assigns a deeper meaning to cooked food – not just a consumption model which ultimately coincides with the word “civilised”.

In the words of Appadurai, at long last we now talk of food as a “highly condensed social fact”, as a “marvellously plastic kind of collective representation”.

Beginning in the eighties more and more papers and studies began to focus on this issue. Important authors, such as Jack Goody and Alan Ward, have devoted in-depth studies to the relationship between cooking and social class, and between flavour and consumption.

People began to reflect on the relationship between eating habits and modernity, and what it actually involves: greater awareness about a balanced diet, the concept of bodily beauty and health, and gender differences.

The most important fact which emerges from a socio-anthropological interpretation of breakfast is that it is the first post-nocturnal meal of the day; while biochemists and doctors highlight its biodynamic aspects, a scholar of social sciences will underscore the fact it is the first meal after the physiological break with consciousness during the night.

This is an aspect emphasised by many primitive cultures which assign special features to the dawn, in part linked to our night-time experiences (cfr. the nocturnal struggle between the sun and the shadows of the night in Mayan culture), and in part to the heritage of light brought by the day (cfr. the myth of Apollo in Greek culture).

So from a socio-anthropological point of view breakfast links the nocturnal dimension of abandonment, unconsciousness, and revelry, with the daytime dimension of awareness, identity, and community. A sort of “no man’s land” which in a few short seconds ferries us from a semi-soporific state to one of complete alertness.

We all know from our own experience that this “half” state is longer for some individuals, and shorter for others. Some individuals can eat a proper meal, others can only just gulp down a coffee, saying they could not possibly eat anything else. Almost as if emerging into a state of awareness is so stressful that food is impossible. So there is a psychological and existential dimension to breakfast, a dimension closely associated with our life events and the seasons: just think of how women “refuse” food when they are pregnant or experience morning sickness, or the “rejection of food” associated with certain depressive states.

Apart from these psycho-existential aspects, cultural paradigms can also be used to interpret breakfast; in turn, these paradigms are often associated with the weather: morning fog in northern Italy has contributed to more substantial breakfasts, with sugars and proteins, while the cool, sunny mornings in the South are conducive to lighter, colourful, vitamin-packed, breakfasts. In Italy a “cappuccino and croissant” – eaten outside the office in a café – have become a mid-morning break to compensate a hasty, meagre breakfast, often eaten at daybreak in the kitchen with the lights on.

Tasty “homemade” breakfasts reminiscent of a caring mother and full-time cook are practically non-existent, killed off by the need to get ready quickly and rush to the office.

In today’s world we not only need to overfeed our children, believing that fat is beautiful (a concept probably leftover from the lean, post-war years, especially in southern Italy), we are also becoming increasingly aware of the nutrients required by our bodies. So the concept of the first meal of the day is linked more to coherent, rational, and pondered choices, rather than to local cultural traditions.

As a result, our job, gender, cultural customs, different realities, local anthropological and territorial traditions, and the existential state of each individual, are closely related to our breakfast habits; they produce a rich and varied mix which increasingly include foods generated by rational globalisation: vitamin-packed and dietetic cereals, several milks that fight intolerance (and yet provide the reassuring smell of whole milk), yogurt, and healthy, tasty probiotics.

This increase in mass-produced foods based on health-oriented criteria is perhaps the one nutritional novelty of the past few years: mass-produced foods are no longer touted as attractive “mixtures” of uncertified origin and composition whose only appeal lies in their artificially induced flavours (e.g., the endless series of chips of any countless number of flavours). On the contrary, mass-produced food now appears keen to satisfy all the requirements recommended by modern dietetics (control, safety, hygiene, dietary intake, and lightness).

### Nutrition and health issues

#### Child and adolescent

Scientific data increasingly shows that breakfast plays an important role in ensuring the good health and wellbeing of an individual. Nevertheless, studies of the eating habits of different populations and age groups reveal how the first meal of the day is often underrated. According to a review of 47 observational studies about eating habits conducted in the United States and Europe, from 10 to 30% of children and adolescents regularly skip breakfast, with a higher percentage among adolescents and the female population [7]. In Spain, for example, the AVENA study showed that 8.6% of female adolescents and 3.5% of male adolescents skip breakfast, with a marked effect of age on the tendency of females to omit breakfast: in particular this figure is 1.7% at age 13 years, against 13.5% at age 17–18.5 years [8]. The HELENA study showed that 9% of European adolescents skip breakfast. In 2008, the survey known as “OKkio alla Salute” showed that in Italy 11% of children skip breakfast, 28% did not eat a qualitatively balanced breakfast, but only ate carbohydrates or proteins [9]. The main reasons for skipping breakfast seem to be related to: lack of time, lack of morning appetite, and, for adolescents, concern about their body weight.

Pearson et al., have reviewed scientific studies assessing how the family influences child or adolescent breakfast consumption [10]. Results indicate that parents not only influence their children’s decision to eat breakfast, but also the food they choose. Furthermore, the study showed how a family’s low socioeconomic status is more related with either skipping breakfast, or eating a nutritionally poor quality breakfast. Overall these studies show that parents are a behavioural model vis-à-vis eating habits: as a result, a positive parental attitude represents a valid tool to instil healthy eating habits compared to either controlling or imposing dietary restrictions [11]. It is also important to allow children to choose the kind of breakfast they prefer from among the ones proposed; this helps to improve their ability to self-regulate food intake and reinforces their consumption habits [12].With regard to the health benefits of eating breakfast we have to consider how, after a long night’s fast, it is the first source of energy we need to go about our cognitive or physical activities. Studies have shown how in children aged 3–11 years, the brain uses 50% of the body’s oxygen. Compared to adults, children have a higher brain/liver weight ratio (1.4-1.6 in children vs. 0.73 in adults), and a 50% higher metabolic activity per unit of weight; the average global cerebral blood flow and the use of oxygen is 1.8-1.3 times greater in children than in adults.

As a result, during the night children require more glucose compared to adults, even though they have lower glycogen stores. Furthermore, children’s smaller muscle mass limits the hepatic gluconeogenic amino acid uptake [13]. Using PET scans, a study of 30 children and adolescents between the ages of 0 and 18 years showed that the use of glucose and the metabolic activity of the brain gradually decreases from age 10 and stabilises around the age of 16 – 18 years [14]. Sugar availability and glycyl metabolism could hence be a factor influencing cognitive performance.

A systematic review of literature, including 45 studies published between 1950 and 2008, showed that breakfast consumption has a positive effect on cognitive performance, in particular on memory and attention span, especially in the second half of the morning when there is a decline in these skills. Some of these studies also highlighted how the positive effect of breakfast on cognitive functions is greater in children whose nutritional status is compromised [15]; several authors also emphasised how a low glycaemic index breakfast positively influences these functions [16-20]. A low glycaemic load breakfast is associated with better memorising skills, as well as with the ability to sustain attention and spend more time doing homework rather than taking to peers or playing. Compared to the composition of macronutrients in a meal, the glycaemic load has been shown to be the factor which effects these functions the most [20]. A healthy breakfast is also associated with an increased ability to solve mathematical problems, and better comprehension while reading and listening. The activity in regions of the brain involved with mental arithmetic was functionally greater in children who had breakfast compared to those whose last meal had been dinner the night before [21]. It is still not perfectly clear how breakfast positively impacts cognitive skills, but presumably involves physiological and behavioural mechanisms. With regard to psychological mechanisms, several studies have concentrated on the role of certain substances associated with glycaemia, such as acetylcholine, insulin, serotonin, glutamate and cortisol, rather than emphasising the direct effect of glucose on cognitive performance (an aspect not shared by all authors). It is possible that central or peripheral mechanisms vary the levels of one or more of these substances and that these variations are involved in the way breakfast influences cognitive performance during the morning [15]. Breakfast consumption enhances an individual’s nutritional status: in fact, several observational studies have shown a relationship between regular breakfast consumption and an increased intake of fibres, calcium, potassium, phosphorous, vitamin A, C, E, B6 and B12, riboflavin, zinc, and iron, as well as a reduced intake of fats and cholesterol [22-24]. Data provided by the Bogalusa Heart Study has shown that most 10-year-olds who skipped breakfast did not get the minimum intake level of vitamin A, B6, D, calcium, magnesium, riboflavin, zinc, and iron [25]. Furthermore, children who skipped breakfast less frequently fulfilled the recommended daily intake of certain types of food, such as fruit and vegetables [26]. Improved nutrient intake is guaranteed chiefly by a breakfast which includes cereals, especially integral cereals. In fact, most ready-to-eat cereals are low in fat and represent a good source of complex carbohydrates and fibres, often fortified with vitamins and minerals.

Breakfast also helps to regulate energy intake during the rest of the day; in fact, due to a heighten feeling of hunger, children and adolescents who regularly skip breakfast tend to eat more food at their next meal, especially high-density, high-fat, food [27]. They also tend to consume greater quantities of added sugar. In particular, a low glycaemic index breakfast (with complex carbohydrates and fibres such as ready-to-eat cereals, bread, rusks and biscuits, preferably integral biscuits) has the greatest influence over energy intake for the rest of the day: in fact, complex carbohydrates influence the release and activity of certain hormones known as incretins, for example gastric inhibitor peptide (GIP), glucagon-like peptide-1 (GLP-1) and colecistocinina; these hormones regulate postprandial satiety and glycaemia [28]. Furthermore, the significant level of proteins and lipids in milk and its derivates, normally eaten during a typical Italian breakfast, not only helps to control the secretion of ghrelin and hence appetite, but also significantly enhances satiety [29].

Warren et al. have demonstrated that preadolescents who eat a low glycaemic index rather than a high glycaemic index breakfast reduce their calorie intake at lunch by approximately 100 kcal [30]. As a result, it is easy to appreciate the relationship (highlighted by several observational studies) between overweight/infant obesity (but also adult obesity) and a reduction in the number of meals, in particular skipping breakfast. The National Health and Nutrition Examination Survey, involving approximately 10,000 children and adolescents in the United States from 1999 and 2006, has shown that skipping breakfast is related to an increase in Body Mass Index (BMI), greater waist circumference, and higher obesity rates.

The study also showed that these anthropometric measurements are smaller in those who eat a cereal breakfast compared to those who eat other kinds of breakfast [31]. A meta-analysis of 3 trials with a total of 2,086 participants showed a BMI increase of 0.78 kg/m^2^ (CI 95% 0.51-1.04) among breakfast skippers.

The E-MOVO project involving 35,000 Dutch secondary school students studied which life habits are most associated with BMI; results show that, compared to alcohol consumption and physical inactivity, breakfast skipping is more closely linked to overweight, especially among younger children [32].

The “OKkio alla Salute” survey also showed a statistically significant relationship between regular breakfast consumption and a prevalence of overweight/obesity: in particular, children who skip breakfast run a higher risk of being overweight or obese compared to their peers who eat a healthy breakfast [9]. Berkeley et al. analysed the annual BMI variations of approximately 14,000 children between the ages of 9 and 14 years. During the initial assessment a relationship between overweight and breakfast skipping was recorded, in line with the studies mentioned above. However, when individuals who were overweight at the start of the study skipped breakfast this caused a reduction in body mass the following year, while the body mass of normal weight children increased. This data suggests that nutritional balance does not only depend on regular breakfasts, but also on the amount and correct composition of the food consumed [33]. Teaching children to eat breakfast, especially a proper, healthy breakfast, is an important weapon not only to fight overweight and obesity, but also indirectly all associated pathologies such as Type 2 mellitus diabetes, cardiovascular diseases, hypertension and osteoarticular diseases.

Furthermore, several studies have highlighted the fact that skipping breakfast negatively affects lipid profile and insulin sensitivity. A study conducted on young, healthy, normal weight women aged between 18 and 39 years showed that skipping breakfast is associated with higher levels of total cholesterol, low density lipoprotein (LDL), and insulinemia [34]. Di Giuseppe et al. assessed the effect of a typical Italian breakfast (milk, coffee, yogurt, croissant, sugar, rusks, biscuits, jam, cereals and honey) on cardiovascular risk factors in a group of 18,177 individuals over 35. Individuals who eat breakfast more frequently were less likely to have a high BMI, abdominal obesity, high blood pressure, high glycaemic index, triglyceridemia, and total cholesterol. Regular breakfast intake is therefore associated with a more favourable metabolic profile. Furthermore, there was a lower prevalence of metabolic syndrome and individual cardiovascular risk. Similar results regarding the effect of breakfast (especially ready-to-eat cereal breakfasts) on lipid profile were also found in studies on adolescents [35,36]. All this points to a significant reduction in cardiovascular diseases and mellitus diabetes amongst regular breakfast eaters [37].

#### Adults and the elderly

Interesting results have emerged from scientific studies analysing several metabolic parameters related to cardiovascular risk in adults aged between 26 and 36 years whose eating habits were also recorded at the outset of the observation, at ages 9 to 15 years [38]. The results show that, compared to those who eat breakfast every day, individuals who skipped breakfast, either during childhood or as an adult, have greater values of Abdominal Circumference (+ 4.63 cm), Insulinemia on an empty stomach (+2.02 mU/L), Total Cholesterol (+0.40 mmol/L or 15 mg/dl) and LDL Cholesterol (+0.40 mmol/L or 15 mg/dl)38. This data shows that in the long run regular breakfast consumption can have positive effects on metabolic parameters related to cardiovascular risk.

A prospective study analysed the data of 13,368 men whose cereal breakfast consumption was assessed using *ad hoc* diet-related questionnaires [39]. During the 16.3 years of observation, 7,267 cases of hypertension were recorded with an incidence of 36.7, 34.0, 31.7, and 29.6 cases annually per 1000 individuals, for consumption respectively of 0, less than 1, from 2 to 6, and 7 or more weekly portions of cereals for breakfast. The risk of developing hypertension was therefore inversely associated with the consumption of these foods; these data were also confirmed by subsequent analyses which used adjusted data for several confounding factors (including age, smoking, BMI, alcohol consumption and level of physical activity), and resulted equal to 1, 0.93, 0.88, and 0.81, from the lowest to the highest category of consumption.

An even greater association was estimated between those who ate integral rather than refined cereals. The results of this study indicate a reduction in the long term risk of developing hypertension for adult men who regularly eat cereals, especially integral cereals, for breakfast [39].

Another prospective study assessed the association between breakfast skipping, daily meal frequency, snack consumption, and the risk of type 2 diabetes, in a group of 29,206 American men. During the 16-year follow-up, 1,994 cases of type 2 diabetes were recorded; results showed that the risk of this disease increased 21% among breakfast skippers and men who ate 1–2 rather than 3 meals a day (relative risk, or equal RR, is respectively 1.25 and 1.00). There was no significant association for consumption of 5–8 meals a day (RR = 0.90). These associations declined after statistical adjustment of Body Mass Index (BMI), indicating that they reflect, at least in part, the effect of eating habits on body weight. The results of this study therefore confirm that regular breakfasts reduce the risk of type 2 diabetes, almost certainly due to less risk of overweight [40].

A controlled study assessed postprandial satiety and energy intake at the next meal (4 hours later) of 34 overweight individuals who ate 2 different isocaloric breakfasts at 2 different times, one with 600 ml of skimmed milk, and the other with the same quantity of fruit juice. Compared to individuals who drunk the fruit juice, the consumption of milk, and hence more proteins, led to increased satiety in the 4 hours after the meal, and a reduced energy intake at lunch [41].

Numerous studies have shown better body weight control in individuals who eat regular breakfasts, especially a cereal-based breakfast [42]. In women, but not men, regular breakfasts are associated with a lower BMI (27.9 vs. 29.4, p = 0,001). A high energy density (ED) breakfast is associated with an increase in ED also during later meals, and greater consumption of fats; instead a lower ED is associated with better food quality at breakfast, especially cereals and fruit.

Despite the fact that extensive scientific data suggests a relationship between diet and cognitive function, so far very few studies have assessed the long-term effect of eating habits on the maintenance of cognitive performance in elderly persons.

One study explored the relationship between the eating habits and global cognitive performance of approximately 3,000 individuals over 45 years of age; 13 years later these individuals were tested to check their verbal memory, attention span, working memory, problem-solving ability, etc. Results show that high intake of integral cereals, fresh milk and dairy products, vegetables, vegetable fats, dry fruit, and fish, improve global cognitive function (scores: 50.1 ± 0.7 vs. 48.9 ± 0.7 comparing the highest quartile of adherence to this diet to the lowest quartile) and verbal memory (49.7 ± 0.4 vs. 48.7 ± 0.4 respectively). This association is particularly strong in individuals with a less-than-average energy intake (<2.490 kcal and <1.810 kcal for men and women respectively). The results of this study support the presence of a relationship between eating habits in adults and maintenance of cognitive function; it suggests that a healthy diet is important even during the presymptomatic stages of cognitive decline [43].

Numerous studies show that the macronutrients, micronutrients, vitamins, minerals, and trace elements normally provided by breakfast, are not compensated by other foods eaten during the rest of the day. Milk should never be missing from the table in the morning and should preferably be combined with high complex carbohydrate foods (ready-to-eat breakfast cereals) which provide a “long-term” energy source and allow us to properly face the working day. Milk is important due to its calcium and phosphorous contents, with essential amino acids. Cereal-based and preferably fortified breakfasts can provide up to 25% of the recommended daily intake of micronutrients. In fact, studies on individuals aged between 5 and 80 years have shown that cereals at breakfast increase the nutritional density of this meal. In Ireland, in particular, a lower incidence of iron deficiency was recorded among women who ate cereals for breakfast.

Other studies confirm that even the circulating levels of thiamine, riboflavin and beta-carotene are favourably influenced by cereal consumption at breakfast, both in children and adults, while the optimal folic acid intake, which depends strictly on the consumption of a fortified breakfast, has been associated, in a population of pregnant Irish women, with a reduced incident of neural tube defects among new-born infants. These data confirm that fortified cereals are an excellent source of micronutrients and make the latter bioavailable. Data regarding regular breakfast consumption and fibre intake have been recorded during epidemiological studies in several countries which considered breakfast cereals an important source of overall daily fibre intake (from 8% to 18% of the total, depending on the author): an important contribution especially for children who normally eat smaller amounts of fibre-rich food. According to a British study, in particular, only regular consumers of integral breakfast cereals achieve the dietary fibre intake recommended by National Guidelines.

The average body composition of an individual changes radically during ageing. Although the BMI value remains constant, there is a tendency to accumulate fat mass between the ages of 30 and 50, and loose a similar amount of muscle mass. Later on, the fat mass and muscle mass begin to decline together, and while in the elderly the fat mass returns to its value at age 30, muscle mass declines even as much as 50-60%. As a result, during old age it is important to protect lean body mass and prevent malnutrition which, in an advanced society such as ours, is not uncommon in the elderly.

More than 50% of the institutionalised geriatric population is actually undernourished or risks malnutrition; likewise, a significant percentage of individuals over 60 are more or less below the Recommended Dietary Allowance (RDA) as concerns specific food components such as vitamin B12, folates, and above all calcium. In fact, on average approximately 90% of geriatric individuals do not assimilate the RDA for total calorie intake, 35% for protein intake, and more than 80% for calcium, vitamin D, and magnesium intake.

It is interesting to note that the percentage of elderly persons who do not assimilate the RDA decreases with an increase in individuals who say they eat regular breakfasts.

In countries where breakfast cereals are fortified with B-group vitamins, their consumption is also associated with a reduction in the haematic levels of homocysteine, an important factor in cardiovascular risk.

So the main nutritional problem affecting the elderly is maintaining a healthy quantitative and qualitative dietary intake. Many mechanisms tend to induce malnutrition by default in the elderly. There is a decline in energy output and physical activity, and a reduction in the sense of smell and taste. Endogenous opioid activity tends to decrease, while there is an increase in neuropeptide values limiting food intake, such as leptin, cholecystokinin (CCK), and peptide YY (PYY). Even gastric emptying is significantly slower compared to younger individuals.

All this contributes to creating a situation in which there is an alteration of the appetite/satiety rhythm, a quicker and more enduring increase in the subjective level of satiety and, on the contrary, a much slower level of appetite after a standard meal.

Apart from the usual attention to fats and sugars, a balanced nutritional meal pattern for the elderly includes a proper supply of water, milk or yogurt, fruit, vegetables, bread, pasta, rice, and cereals and regular vitamin B12, vitamin D, and calcium supplements.

More specifically, one should not forget that it is very important for an elderly person to have a good breakfast which includes milk and yogurt.

The data presented in the Seneca study unfortunately shows that in Italy the average percentage of calorie intake at breakfast is limited and lower compared to other European countries.

### Bio-psycho-social point of view

Scientific literature shows that breakfast in pre-school and school children has a direct effect on psychological functioning. In particular, research has focused on the relationship between eating breakfast and cognitive performance, in other words on the ability to perform all tasks which include memory, concentration, learning, and the ability to make decisions based on available information. Breakfast activates at least two psychophysical mechanisms to facilitate a child’s cognitive performance. In the short term breakfast stops depletion of the nutrients needed for the central nervous system to function properly. Studies on children aged between 3 and 11 years have shown that the brain uses more than 50% of the oxygen employed by the whole body. Breakfast provides the basic micro and macronutrients required for this process and enables glucose oxidation. Skipping breakfast may therefore momentarily reduce the availability of glucose or nutrients required by neurotransmitters to allow the nervous system to function properly.

Breakfast improves the overall nutritional intake of a diet, which is an important determinant in efficient cognitive processes. Not unsurprisingly, these are the reasons why the metabolic effects of skipping breakfast and prolonging the fasting period until dinner can negatively influence a child’s scholastic performance [44]. On the contrary, regular breakfast consumption is associated with improvement in memorisation, attention span, concentration, the ability to solve mathematical problems, and comprehension during reading and listening; it is also associated with a reduction in school absences, arriving late for lessons, and improved scholastic involvement [45].

This interpretation has methodological deficits. Many studies assess children’s cognitive performance before and after breakfast, but few studies have assessed the long term effects of this meal on cognitive functioning [44]. Furthermore, only a handful of studies have properly assessed other important factors, such as children’s physical exercise, their socioeconomic status, and family environment [15].

A less studied problem is the relationship between breakfast and a child’s mood. The results of these infrequent studies show that children and adolescents who regularly eat breakfast have more positive feelings, better concentration, and less negative feelings [46]. If confirmed by further research, there are several ways in which we could interpret these data. Firstly, on an emotional-relational level, we should not underestimate the effect of a family moment on a child’s mood in the early morning, before family members separate and go their own way to face the day. Secondly, on a more metabolic level, the fact that children who skip breakfast have to tackle their first daily tasks without an energy reserve, and this certainly does not help their mood. Thirdly, on a psycho-biological level, the composition of the macronutrients eaten during breakfast can influence mood by acting on processes of neurotransmission [46].

### Teacher and parental educational role

Thanks to their educational role, teachers can facilitate child/food interaction. Health education projects in schools appear to be the winning solution to achieve long-term results, as against the irregular work of an outsider who, although backed by the teacher, is less successful than a structured daily school routine. This is possible because the teacher’s pedagogical role makes him/her the person tasked with the child’s extrafamilial education. Scientific literature has emphasised how even in infancy children should be given the right cognisant and vocational tools to allow them to make their own health protection choices, or on the contrary express criticism.

It is important children be aware they are responsible for their own health, and that the school and the family support them. The proposed educational programme is based on body awareness, growth, food, habits, and the importance of breakfast.

Breakfast can be used as an excellent opportunity to launch and develop educational strategies to establish and enhance a correct approach to food and eating. Pedagogically speaking, this opportunity is based on several important factors, first and foremost setting an example. A parent who eats well - in other words, who avoids snacks between meals, who puts lots of fruit and seasonal vegetables on the table, and who does not purchase ready-to-eat meals, and prepares meals carefully - will probably transmit the same dietary model to his/her children who tend to emulate the good and bad examples set by their parents.

Never treat food as a reward: we eat to nourish and not to console or gratify ourselves. If we teach children this truth and avoid rewarding them with food, they will be more inclined to assign food the right role. This does not mean we should not give them what they like, but simply that eagerly eating (healthy) food should be part of our eating habits.

Food is not a game: children should be used to sitting at table to eat, without being distracted by technology or the mass media. No tricks should be used to distract them and force them to eat their food without even realising what they are swallowing. Instead, they should be taught to appreciate their food, even from a very early age. Games and creative activities can be important in educational/training programmes as a tactic to approach nutrition, a way to learn about food; the child will be able to re-elaborate and turn the food and its transformations into a personal image; he will become the protagonist of a pleasant environment and give free rein to his feelings, relationships, and tangible experiences by acquiring and enhancing good, long-lasting, eating habits and behaviour.

Finally, one should remember how important habits are: children are creatures of habit, so if they adopt certain habits they will be less likely to eat between meals, and instead be ready to eat at mealtimes. Good eating habits should be acquired at a very early age, because a natural approach to food that continues during later periods of growth is behind a healthy eating model.

All this becomes “memory” and children can use it to remember past experiences and develop positive attitudes and a correct behavioural approach to their health. At the same time, it is important to avoid a “moralistic” attitude if we want a child to adopt a happy and relaxed approach to his choice of lifestyle, fully aware of the importance of his/her own life and each individuals’ personal traits.

As a result, breakfast is the basis for an educational programme dedicated to dietary well-being. It is therefore important to help children acquire the information they need to consciously choose the food they require to grow and maintain good health and well-being, emphasising the need for a regular breakfast every day and its effect on our personal life, tastes, feelings, sociality, and environment; all these actions are aimed at improving our eating habits and turning the early morning into a pleasant and “delicious/enjoyable” moment of peace and quiet so that we can optimistically face waking up.

### Summary

The aim of this paper was to analyse breakfast-related issues based on a multidisciplinary approach with input by specialists from different fields of learning.

Our eating habits depend on a culture that vary in time and space, as well as on our own personal choices. Of all our meals, none varies more than breakfast.

Breakfast is the first opportunity we have to intake the energy we need to perform our daily activities. Correct nutritional intake improves long term cognitive performance, regulates energy intake during the other meals of the day (by reducing total daily calorie and lipid intake), and increases the intake of fibres, vitamins and minerals; this improves our nutritional status throughout life and has a positive effect on the prevention of chronic-degenerative diseases such as overweight, obesity, hypertension, and Type 2 diabetes.

Although all reported data underline the important role that breakfast plays in maintaining the health and well-being of an individual, epidemiological data from industrialised countries reveal that many individuals either eat a nutritionally unhealthy breakfast or skip it completely. Not enough time and a lack of appetite upon awakening are the main reasons cited; in addition, adolescents incorrectly believe that skipping breakfast can help them control their weight, while the elderly suffer from an alteration of their hunger/appetite rhythm.

We need to adopt a systematic approach to these problems including everything that affects our relationship with breakfast.

## Competing interests

The authors declare that they have no competing interests.

## Authors’ contributions

GVZ and CM critically revised the entire manuscript. AA, LC, GC, GDL, DD, GD, LF, FL, EM, PM, GM, LM, MM, GR, MS drafted the manuscript. All authors read and approved the final manuscript.

## References

[B1] Montanari M Alimentazione e cultura nel Medioevo Monastic rules about food 1989 Roma-Bari: Laterza 63

[B2] Aymard M Grignon C Sabban F Maison des sciences de l‘homme / Institut National de la Recherche Agronomique Le temps de manger Alimentation, emploi du temps et rythmes sociaux 1993 Paris: Institut National de la Recherche Agronomique

[B3] Baretti G Gl’Italiani o sia Relazione degli usi e costumi d’Italia Capitolo XXV: Vita quotidiana degl’Italiani – Loro nutrimento ordinario – Necessità del ghiaccio in Italia 1818 Milan: Pirotta 209 2014

[B4] Montanari M Nuovo Convivio. Storia e cultura dei piaceri della tavola nell’Età moderna 1991 Rome-Bari: Laterza 315 316

[B5] Camporesi P Il brodo indiano. Edonismo ed esotismo nel Settecento 1990 Milan: Garzanti

[B6] Montanari M La fame e l’abbondanza. Storia dell’alimentazione in Europa 1993 Rome-Bari: Laterza 153 159

[B7] Rampersaud GC Pereira MA Girard B Adams J Metzl JD Breakfast habits, nutritional status, body weight, and academic performance in children and adolescents J Am Diet Assoc 2005 105 743 760 10.1016/j.jada.2005.02.007 15883552

[B8] Moreno LA Mesana MI Fleta J Ruiz JR González-Gross M Sarría A Marcos A Bueno M AVENA Study Group Overweight, obesity and body fat composition in Spanish adolescents. The AVENA study Ann Nutr Metab 2005 2 71 76 15802900 10.1159/000084738

[B9] Spinelli A Lamberti A Baglio G Andreozzi S Galeone D OKkio alla SALUTE: sistema di sorveglianza su alimentazione e attività fisica nei bambini della scuola primaria. Risultati 2008 Rapporti ISTISAN 2009 09 24

[B10] Pearson N Biddle SJ Gorely T Family correlates of breakfast consumption among children and adolescents. A systematic review Appetite 2009 52 1 7 10.1016/j.appet.2008.08.006 18789364

[B11] Scaglioni S Salvioni M Galimberti C Influence of parental attitudes in the development of children eating behavior Br J Nutr 2008 99 1 22 25 10.1017/S0007114508892471 18257948

[B12] Birch LL Fisher JO Development of eating behaviors among children and adolescents Pediatrics 1998 101 539 549 12224660

[B13] Sokoloff L Circulation and energy metabolism of the brain. Basic Neurochemistry 1981 1 Boston: Little Brown 471 495

[B14] Chugani HT A critical period of brain development: studies of cerebral glucose utilization with PET Prev Med 1998 27 184 188 10.1006/pmed.1998.0274 9578992

[B15] Hoyland A Dye L Lawton CL A systematic review of the effect of breakfast on the cognitive performance of children and adolescents Nutr Res Rev 2009 22 220 243 19930787 10.1017/S0954422409990175

[B16] Ingwersen J Defeyter MA Kennedy DO Wesnes KA Scholey AB A low glycaemic index breakfast cereal preferentially prevents children’s cognitive performance from declining throughout the morning Appetite 2007 49 240 244 10.1016/j.appet.2006.06.009 17224202

[B17] Mahoney CR Taylor HA Kanarek RB Samuel P Effect of breakfast composition on cognitive processes in elementary school children Physiol Behav 2005 85 635 645 10.1016/j.physbeh.2005.06.023 16085130

[B18] Wesnes KA Pincock C Richardson D Helm G Hails S Breakfast reduces declines in attention and memory over the morning in schoolchildren Appetite 2003 41 329 331 10.1016/j.appet.2003.08.009 14637332

[B19] Benton D Ruffin MP Lassel T Nabb S Messaoud N Vinoy S Desor D Lang V The delivery rate of dietary carbohydrates affects cognitive performance in both rats and humans Psychopharmacology (Berl) 2003 166 86 90 12488949 10.1007/s00213-002-1334-5

[B20] Benton D Jarvis M Williams C The influence of the glycaemic load of breakfast on the behaviour of children in school Physiol Behav 2007 92 717 724 10.1016/j.physbeh.2007.05.065 17617427

[B21] Pivik RT Tennal KB Chapman SD Gu Y Eating breakfast enhances the efficiency of neural networks engaged during mental arithmetic in school-aged children Physiol Behav 2012 106 548 555 10.1016/j.physbeh.2012.03.034 22504496

[B22] Nicklas TA Myers L Reger C Beech B Berenson GS Impact of breakfast consumption on nutritional adequacy of the diets of young adults in Bogalusa, Louisiana: ethnic and gender contrasts J Am Diet Assoc 1998 98 1432 1438 10.1016/S0002-8223(98)00325-3 9850113

[B23] Kleemola P Puska P Vartiainen E Roos E Luoto R Ehnholm C The effect of breakfast cereal on diet and serum cholesterol: a randomized trial in North Karelia, Finland Eur J Clin Nutr 1999 53 716 721 10.1038/sj.ejcn.1600849 10509768

[B24] Gibson SA O’Sullivan KR Breakfast cereal consumption patterns and nutrient intakes of British schoolchildren J R Soc Health 1995 115 366 370 10.1177/146642409511500608 8568785

[B25] Nicklas TA Dietary studies of children: the Bogalusa Heart Study experience J Am Diet Assoc 1995 95 1127 1133 10.1016/S0002-8223(95)00305-3 7560684

[B26] Utter J Scragg R Mhurchu CN Schaaf D At-home breakfast consumption among New Zealand children: associations with body mass index and related nutrition behaviors J Am Diet Assoc 2007 107 570 576 10.1016/j.jada.2007.01.010 17383261

[B27] Nicklas TA Yang SJ Baranowski T Zakeri I Berenson G Eating patterns and obesity in children. The Bogalusa Heart Study Am J Prev Med 2003 25 9 16 10.1016/S0749-3797(03)00098-9 12818304

[B28] Bornet FR Jardy-Gennetier AE Jacquet N Stowell J Glycaemic response to foods: impact on satiety and long-term weight regulation Appetite 2007 49 535 553 10.1016/j.appet.2007.04.006 17610996

[B29] Foster-Schubert KE Overduin J Prudom CE Liu J Callahan HS Gaylinn BD Thorner MO Cummings DE Acyl and total ghrelin are suppressed strongly by ingested proteins, weakly by lipids, and biphasically by carbohydrates J Clin Endocrinol Metab 2008 93 1971 1979 10.1210/jc.2007-2289 18198223 PMC2386677

[B30] Warren JM Henry CJ Simonite V Low glycaemic index breakfasts and reduced food intake in preadolescent children Pediatrics 2003 112 e414 10.1542/peds.112.5.e414 14595085

[B31] Deshmukh-Taskar PR Nicklas TA O’Neil C Keast D Radcliffe JD Cho S The relationship of breakfast skipping and type of breakfast consumption with nutrient intake and weight status in children and adolescents: the National Health and Nutrition Examination Survey 1999–2006 J Am Diet Assoc 2010 110 869 878 10.1016/j.jada.2010.03.023 20497776

[B32] Croezen S Visscher TL Ter Bogt NC Veling ML Haveman-Nies A Skipping breakfast, alcohol consumption and physical inactivity as risk factors for overweight and obesity in adolescents: results of the E-MOVO project Eur J Clin Nutr 2009 63 405 412 10.1038/sj.ejcn.1602950 18043703

[B33] Berkery CS Rockett HR Gilman MW Field AE Colditz GA Longitudinal study of skipping breakfast and weight change in adolescents Int J Obes Relat Metab Disord 2003 27 1258 1266 10.1038/sj.ijo.0802402 14513075

[B34] Farshchi HR Taylor AM Macdonald IA Deleterious effects of omitting breakfast on insulin sensitivity and fasting lipid profiles in healthy lean women Am J Clin Nutr 2005 81 388 396 15699226 10.1093/ajcn.81.2.388

[B35] Albertson AM Affenito SG Bauserman R Holschuh NM Eldridge AL Barton BA The relationship of ready-to-eat cereal consumption to nutrient intake, blood lipids, and body mass index of children as they age through adolescence J Am Diet Assoc 2009 109 1557 1565 10.1016/j.jada.2009.06.363 19699835

[B36] Hallström L Labayen I Ruiz JR Patterson E Vereecken CA Breidenassel C Gottrand F Huybrechts I Manios Y Mistura L Widhalm K Kondaki K Moreno LA Sjöström M Breakfast consumption and CVD risk factors in European adolescents: the HELENA (Healthy Lifestyle in Europe by Nutrition in Adolescence) Study Public Health Nutr 2013 16 1296 1305 10.1017/S1368980012000973 22494882 PMC10271783

[B37] Kochar J Djoussé L Gaziano JM Breakfast cereals and risk of type 2 diabetes in the Physicians’ Health Study I Obesity (Silver Spring) 2007 15 3039 3044 10.1038/oby.2007.362 18198313

[B38] Smith KJ Gall SL McNaughton SA Blizzard L Dwyer T Venn AJ Skipping breakfast: longitudinal associations with cardiometabolic risk factors in the Childhood Determinants of Adult Health Study Am J Clin Nutr 2010 92 1316 1325 10.3945/ajcn.2010.30101 20926520

[B39] Kochar J Gaziano JM Djoussé L Breakfast cereals and risk of hypertension Clin Nutr 2012 31 89 92 10.1016/j.clnu.2011.08.001 21868140 PMC3289098

[B40] Mekary RA Giovannucci E Willett WC Van Dam RM Hu FB Eating patterns and type 2 diabetes risk in men: breakfast omission, eating frequency, and snacking Am J Clin Nutr 2012 95 1182 1189 10.3945/ajcn.111.028209 22456660 PMC3325839

[B41] Dove ER Hodgson JM Puddey IB Beilin LJ Lee YP Mori TA Skim milk compared with a fruit drink acutely reduces appetite and energy intake in overweight men and women Am J Clin Nutr 2009 90 70 75 10.3945/ajcn.2008.27411 19474132

[B42] Kant AK Andon MB Angelopoulos TJ Rippe JM Association of breakfast energy density with diet quality and body mass index in American adults: National Health and Nutrition Examination Surveys, 1999–2004 Am J Clin Nutr 2008 88 1396 1404 18996877 10.3945/ajcn.2008.26171

[B43] Péneau S Galan P Jeandel C Ferry M Andreeva V Hercberg S Kesse-Guyot E SU.VI.MAX 2 Research Group: fruit and vegetable intake and cognitive function in the SU.VI.MAX 2 prospective study Am J Clin Nutr 2011 94 1295 1303 10.3945/ajcn.111.014712 21955649

[B44] Widenhorn-Müller K Hille K Klenk J Weiland U Influence of having breakfast on cognitive performance and mood in 13- to 20-year-old high school students: results of a crossover trial Pediatrics 2008 122 279 284 10.1542/peds.2007-0944 18676544

[B45] Basch CE Breakfast and the achievement gap among urban minority youth J Sch Health 2011 81 635 640 10.1111/j.1746-1561.2011.00638.x 21923876

[B46] Leigh Gibson E Green MW Nutritional influences on cognitive function: mechanisms of susceptibility Nutr Res Rev 2002 15 169 206 10.1079/NRR200131 19087403

